# The landscape of microRNA interaction annotation: analysis of three rare disorders as a case study

**DOI:** 10.1093/database/baad066

**Published:** 2023-10-11

**Authors:** Simona Panni, Kalpana Panneerselvam, Pablo Porras, Margaret Duesbury, Livia Perfetto, Luana Licata, Henning Hermjakob, Sandra Orchard

**Affiliations:** Dipartimento di Biologia Ecologia e Scienze della Terra, Università della Calabria, Rende 87036, Italy; European Molecular Biology Laboratory, European Bioinformatics Institute (EMBL-EBI), Wellcome Genome Campus Hinxton, Cambridge CB10 1SD, UK; European Molecular Biology Laboratory, European Bioinformatics Institute (EMBL-EBI), Wellcome Genome Campus Hinxton, Cambridge CB10 1SD, UK; Astra Zeneca, Data Office, Data Science and AI, UK Academy House, 136 Hills Road, Cambridge CB2 8PA, UK; European Molecular Biology Laboratory, European Bioinformatics Institute (EMBL-EBI), Wellcome Genome Campus Hinxton, Cambridge CB10 1SD, UK; Department of Biology and Biotechnologies “Charles Darwin”, La Sapienza University, Rome, Italy; Department of Biology, University of Tor Vergata, Rome, Italy; European Molecular Biology Laboratory, European Bioinformatics Institute (EMBL-EBI), Wellcome Genome Campus Hinxton, Cambridge CB10 1SD, UK; European Molecular Biology Laboratory, European Bioinformatics Institute (EMBL-EBI), Wellcome Genome Campus Hinxton, Cambridge CB10 1SD, UK

## Abstract

In recent years, a huge amount of data on ncRNA interactions has been described in scientific papers and databases. Although considerable effort has been made to annotate the available knowledge in public repositories, there are still significant discrepancies in how different resources capture and interpret data on ncRNA functional and physical associations. In the present paper, we present a collection of microRNA–mRNA interactions annotated from the scientific literature following recognized standard criteria and focused on microRNAs, which regulate genes associated with rare diseases as a case study. The list of protein-coding genes with a known role in specific rare diseases was retrieved from the Genome England PanelApp, and associated microRNA–mRNA interactions were annotated in the IntAct database and compared with other datasets. RNAcentral identifiers were used for unambiguous, stable identification of ncRNAs. The information about the interaction was enhanced by a detailed description of the cell types and experimental conditions, providing a computer-interpretable summary of the published data, integrated with the huge amount of protein interactions already gathered in the database. Furthermore, for each interaction, the binding sites of the microRNA are precisely mapped on a well-defined mRNA transcript of the target gene. This information is crucial to conceive and design optimal microRNA mimics or inhibitors to interfere *in vivo* with a deregulated process. As these approaches become more feasible, high-quality, reliable networks of microRNA interactions are needed to help, for instance, in the selection of the best target to be inhibited and to predict potential secondary off-target effects.

**Database URL:**  https://www.ebi.ac.uk/intact

Key points

- Rare diseases are considered one of the main public health problems.
- Little is known about the involvement of microRNAs in rare diseases.
- MicroRNAs exert their function by regulating target genes.
- We propose a method to identify microRNAs potentially involved in rare diseases.
- We have collected the interactions in the IntAct database and compared them with other collections.

## Introduction

**Figure 1.**
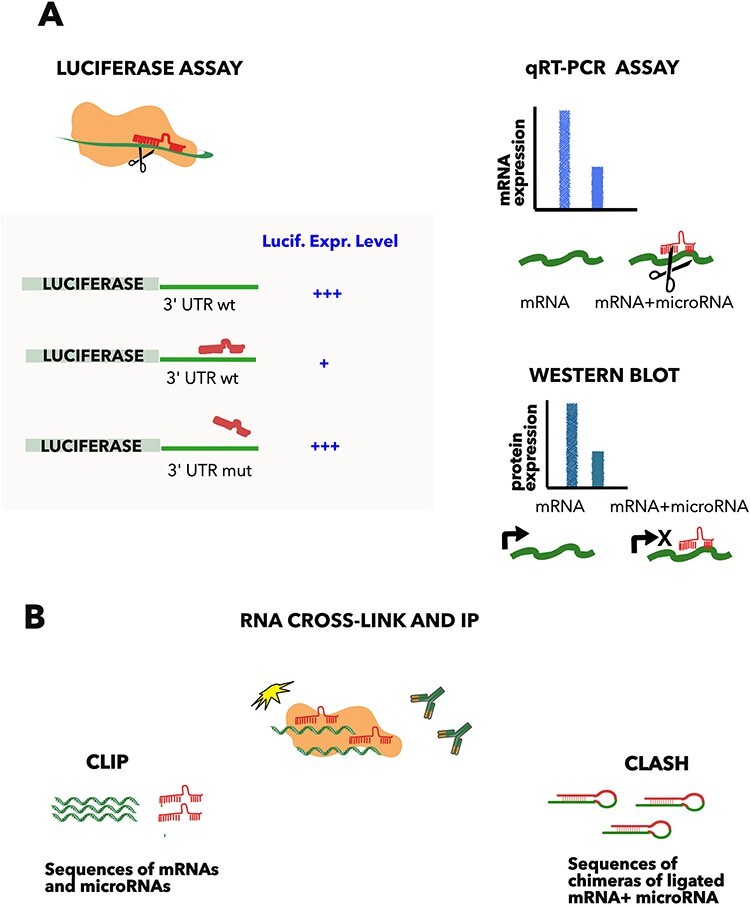
Methods commonly used to detect microRNA–mRNA interactions. (A) Luciferase assay comprises the fusion of the luciferase gene to the 3ʹ UTR of the microRNA target gene and the subsequent transfection of the construct with or without the microRNA. The approach demonstrates the direct interaction, compared to a mutated copy of the predicted complementary region, which acts as a negative control. Quantification of the mRNA (qRT-PCR) or protein level (western blot) helps to distinguish between the mRNA degradation and the translation inhibition, but is not proof of an interaction when used in isolation. (B) CLIP and CLASH. Cross-linking and immunoprecipitation of a RNA binding protein (such as AGO2), followed by RNA sequencing, allows the determination of all the RNAs bound to the protein. These approaches demonstrate direct RNA–RNA binding, if the two interacting RNAs are ligated in a hybrid before sequencing (CLASH (9)).

In recent years, it has become increasingly clear that complex and dynamic interactions between ncRNA molecules or ncRNAs and proteins contribute to virtually any biological process. MicroRNAs are probably the best characterized ncRNAs, as they can be identified using bioinformatics approaches, thanks to the conserved hairpin shape of precursor transcripts, and their possible targets can be predicted (1). MicroRNAs regulate gene expression by guiding the RNA-induced silencing complex (RISC) to those mRNAs, which display sequences complementary to the microRNA ‘seed’, i.e. nucleotides 2–7 from the 5ʹ end (2, 3). Predicted and verified microRNAs from more than 270 species are annotated in miRbase (4), with their precursor and mature sequences and other useful information. The latest release contains 1917 human hairpin precursors and 2654 human mature sequences, of which 26% are high-confidence microRNAs (4). Several microRNA precursors encode two active microRNAs, named 3p and 5p, respectively, which recognize different targets. Identifying the exact function for each miRNA is challenging and time-consuming, and only a subset of them have been characterized. As mentioned earlier, it is possible to predict all possible targets for any microRNA, based on the sequence complementarity, site conservation and other features (1, 5). However, these methods provide a list of hundreds of targets for each miRNA, many of which are not bona fide interactors and need to be experimentally verified (6, 7). To this aim, several techniques have been developed, for low- and high-throughput interaction detection, which we briefly summarize here. One of the most frequently used assays is the luciferase reporter assay, which is an adaptation of the homonymous test used to identify the regulatory regions on DNA promoters (Figure 1). The method permits not only verification of the interaction but also the mapping of the precise binding site on the mRNA, through mutagenesis of the predicted sequence (8). QRT-PCR and western blot allow the quantification of the mRNA and protein levels, respectively, following microRNA over-expression, and can be used to discriminate between mRNA degradation and translation inhibition, which are the two possible results of microRNA interaction (Figure 1). Pull-down and immunoprecipitation approaches, commonly used to demonstrate protein binding, have been adapted to demonstrate microRNA interactions. Cross-linking ligation and sequencing of hybrids (CLASH) allows high-throughput identification of RNA–RNA interactions. In this approach, the RNA is cross-linked to the bait protein, and, after immunoprecipitation, the ends of co-precipitated, interacting RNAs are ligated together and sequenced to identify the couples (9). Other high-throughput sequencing approaches of immunoprecipitated RNAs, after cross-linking to proteins of the RISC complex (crosslinking immunoprecipitation (CLIP)-seq, photoactivatable ribonucleoside-enhanced crosslinking and immunoprecipitation (PAR-CLIP), etc.), provide datasets of potential RNA–RNA interactions (10), largely improving the performance of the binding predictors, although specific couples of miRNA–mRNA should be further verified (11). While the number of publications describing microRNA interactions is constantly increasing, several databases have begun to collect them, often including high-throughput indirect or weak evidence. It is worth noting that miRTarBase (12) and RAID (13, 14) allow filtering of collected ncRNA associations, to reduce false-positive rates when selecting ‘strong evidence’ interactions. The inclusion of potentially erroneous targets in network analysis may result in misleading data interpretation, as extensively discussed in (15). The UCL Functional Gene Annotation group has focused on the gene ontology annotation of human microRNAs collecting a set of highly reliable microRNA–mRNA pairs (15, 16).

Since 2002, the Human Proteome Organization - Proteomic Standard Iniziative has provided a standardized annotation system for molecular interactions and has defined the minimal information requirements and the syntax of terms used to describe an interaction experiment Minimum Information for Molecular Interaction (17), approved by members of the International Molecular Exchange (IMEx) Consortium (18, 19). Common guidelines help to elude false positives and increase the level of details and contextual information captured when describing molecules, such as protein binding (20). The IntAct database, which is a member of the Consortium, has expanded its activities into the annotation of ncRNA interactions, initially focusing on *Saccharomyces cerevisiae* ncRNAs (21) and subsequently on mammalian ncRNAs. In this paper, we present the collection of microRNA–mRNA interactions annotated in IntAct and we integrate and compare it with other resources. In particular, we have focused on the microRNAs that regulate genes associated with rare diseases, in order to provide information potentially useful for the development of new drugs. A rare disease is a health condition that affects a minority of people compared with other diseases that are prevalent in the population (22). Despite the increased focus on these diseases in the last few years, very little is known about microRNAs involved in rare diseases and their collection may help to gain insights into pathological mechanisms. The interactions can be downloaded from the IntAct database (https://www.ebi.ac.uk/intact/) and also from the RNA central website (https://rnacentral.org/).

## Materials and methods

### Data collection and deposition

MicroRNA–mRNA interactions were manually annotated in the IntAct database, according to the curation standards established by the IMEx Consortium. We retrieved relevant papers by searching the literature with text-mining seeking for the term ‘microRNA’ (or synonymous) AND a specific gene name in the abstract and luciferase assay or RNA-Immunoprecipitation or CLASH in the methods. The collected papers were manually filtered to identify those relevant for the curation process. More than 260 papers were selected, and the interactions were annotated according to approved hierarchical terms [Controlled Vocabulary terms, originally created to annotate protein–protein interactions (23), then expanded to other molecular interactions ((21), present work)]. When more evidence supports an edge, a score of reliability is generated, which helps the user to interpret the network (24).

**Figure 2.**
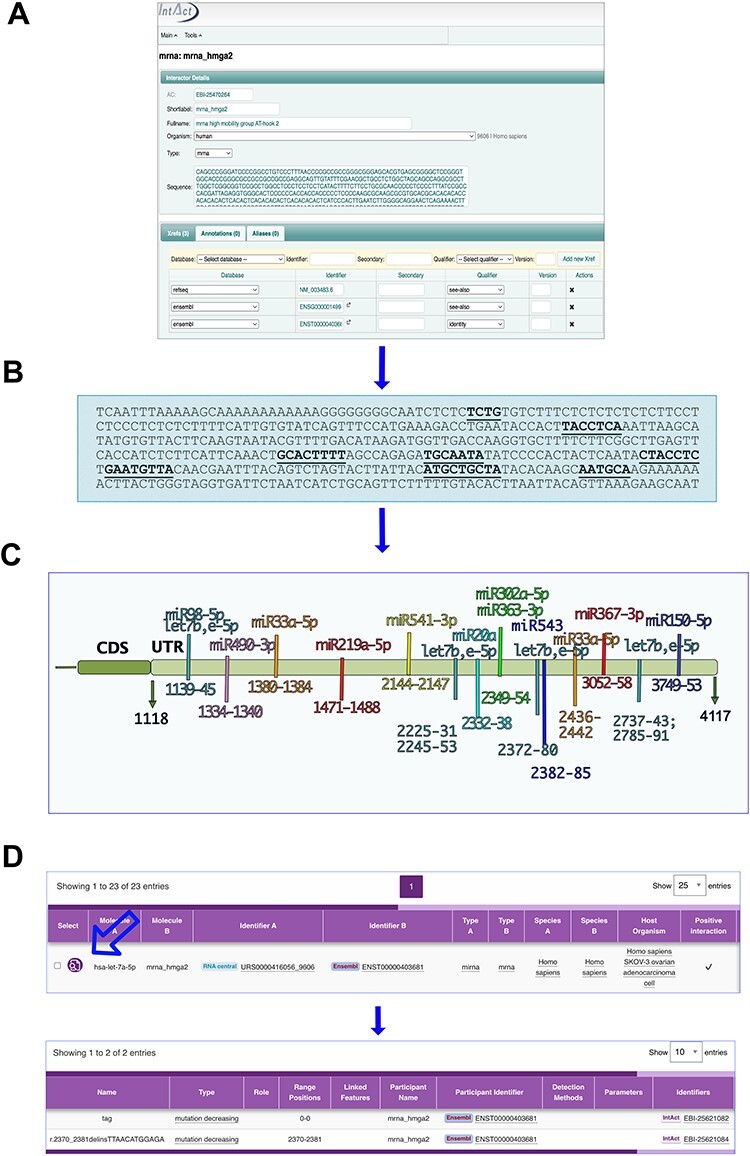
MicroRNA binding sites on mRNA_hmga2 entry. (A) and (B) The annotation process. (A) mRNA_hmga2 entry in IntAct is annotated with the sequence and ID of the main transcript of the gene (identified by GIFT), together with the Ensembl gene ID and a RefSeq. (B) Mutations affecting the interactions are mapped on the transcript sequence. (C) Hmga2-3ʹUTR regions identified as necessary for the microRNA binding. (D) The interaction viewer in the webpage: the arrow indicates the lens to select to see the features.

Genes associated with rare diseases were prioritized for annotation. The list of these genes was generated according to green panels from GenomeEngland PanelApp for the following diseases: aniridia, fanconi anaemia, autism, cakut, cerebral folate deficiency, epidermolysis bullosa, familial Hirschsprung disease, growth failure in early childhood (GFEC), early onset disease (EOD) and mitochondrial disorders (MD). These last three presented a higher number of genes regulated by microRNAs and were used in the subsequent analysis. The microRNAs are identified with RNAcentral IDs and a short label, which specify the strand of the mature microRNA (25).The database can be queried with Ensembl transcript IDs (https://www.ensembl.org/index.html) (26) or gene common names (then selecting mRNA) or with mRNA common names (mrna_name), as well as with the microRNA name or ID. To retrieve information on mutagenesis analysis, in order to map the binding nucleotides, from the IntAct web page, click on the lens in the ‘Select’ column of the displayed interactions and then select ‘Features’ in the following web page (Figure 2D and (27)). Data are also linked to RNA central (25) in the microRNA entry.

### Network analysis and database comparison

The molecular interaction network of microRNA–mRNA interactions was downloaded from IntAct using the IntAct app (28). To build disease-specific networks, the list of Ensembl transcript IDs was used. Networks were built and analysed using Cytoscape (29).

For comparison with other databases, miRTarBase and RAID were chosen to enable the selection of strong evidences of direct binding, comparable to those annotated in IntAct. The data were downloaded, IDs were uniformed, and the tables were uploaded in Cytoscape to build integrated and intersected networks.

### Functional enrichment analysis

For the ‘Biological Processes’ enrichment analysis of microRNA targets, each transcript ID was converted to the common gene name. The tables were imported in Cytoscape to build the network, and the BINGO tool (30) was used to perform the enrichment with 0.003 as the significance level for GFEC and EOD-related genes, while 0.05 was set for mitochondrial disorders (MD) because there was no enrichment at lower values.

### Statistical analysis

#### Correlation analysis

A table was generated containing the number of interacting microRNAs for each gene in each of the three databases (Supplementary Table 1). Pearson's correlation coefficient was calculated using the ggpairs function in the GGally R package (31, 32).

#### Venn Diagram

Intersections among datasets were outlined by ggVennDiagram library (33) in R (32), and Jaccard index was calculated as follows: $J(A,B) = \frac{{A\, \cap \,B}}{{A\,\cup \,B}}$ (34).

## Results

### Building microRNA networks in IntAct

The main molecular function of microRNAs is to downregulate the target gene by binding to the mRNA through few complementary nucleotides located at 5ʹ of the microRNA and commonly (although not always) at the 3ʹ UTR of the messenger RNA. As discussed in the Introduction section, several experimental approaches have been developed to investigate microRNA function, but only a subset provides clear evidence of microRNA binding to mRNA, while discrimination between direct binding and causal interaction is crucial for the design of interfering drugs.

The partners of the IMEx Consortium of interaction databases (18, 19) have agreed to consider luciferase assay as a proof of RNA–RNA interaction, when validated using mutagenesis analysis (Figure 1). Papers describing microRNA–mRNA interactions validated using the above-mentioned methods were selected from the literature and annotated in the IntAct database. As the interaction with mRNA results in the downregulation of the expression, through the mRNA degradation, or in the inhibition of the translation, the microRNA is featured as a ‘regulator’ and a causality statement was used to annotate its effect.

Most of the repositories which collect microRNA interactions identify the targets with the gene name or ID. However, microRNAs bind to messenger RNAs and, in order to map the binding sites on the target, it is necessary to refer to a specific transcript. To this aim, it is worth noting that each gene produces several messengers by alternative splicing of the precursor transcript. The number of the collected transcripts is destined to increase as new sequences will be produced from different cell types and stages. To avoid ambiguities, we have therefore created one full-detailed entry for each target gene, cross-referred it with its Ensembl transcript ID (26) and, whenever possible, have mapped all the annotated interactions on this entry. Figure 2 shows how the entries are created with their sequence. For human transcripts, among the possible transcripts listed in the Ensembl database (26), we have selected the reference shown in the GIFT curation tool (https://www.ebi.ac.uk/gifts/). Each mutation is annotated with a short label compliant with Human Genome Variation Society recommendations (https://varnomen.hgvs.org/), which identifies the nucleotide position and the mutated residues, and the information is displayed in the IntAct web interface (Figure 2D).

**Figure 3.**
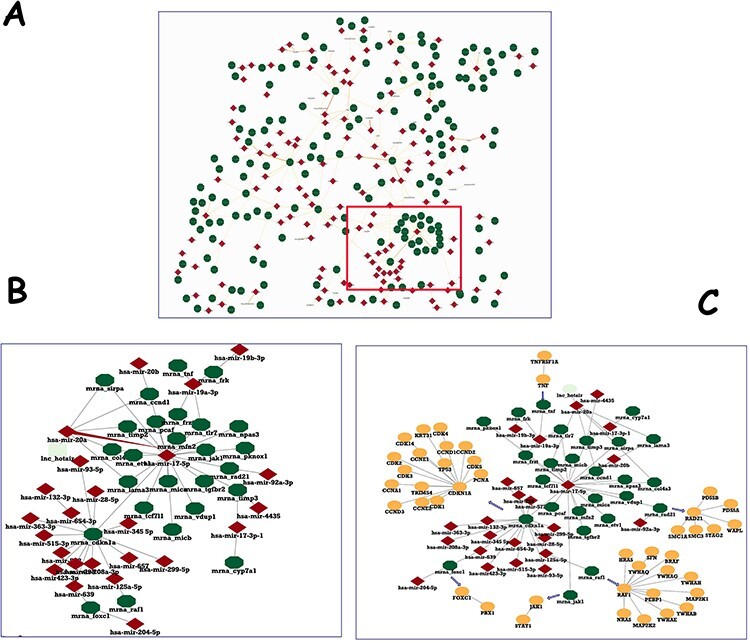
Human microRNA network in IntAct. (A) The section of the human microRNA network, showing hubs of both entry types. (B) Enlargement of a detail to show two nodes and their interactors. (C) PPI network for the genes targeted by hsa-mir-17-5p was downloaded and filtered to a molecular interaction (MI) score (24) of ⋝0.7 and merged with microRNA–mRNA interactions.

We have mainly annotated human interactions, although mouse entries are also represented. Figure 3A shows a subset of the microRNA–mRNA human interaction network. Similar to protein-protein interaction (PPI) networks, few nodes of both molecular types behave as hubs, presenting a number of interactions above average. The network detail blown up in Figure 3B shows two connected hubs: the microRNA hsa-mir-17-5p and the mRNA_cdkn1a.

The IntAct database contains thousands of human protein interactions, each with a reliability score, so it is possible to investigate the protein complexes affected by the microRNA regulation and the connections between the protein products of co-regulated genes. Figure 3C shows some high-reliable interactors of hsa-mir-17-5p targets that may be deregulated by mimics or inhibitors of the microRNA.

### MicroRNA regulation of rare disease-associated genes

While the amount of microRNA interaction information is increasing continuously, very little is known about microRNAs involved in rare diseases.

Until recently, there has been limited research on the molecular mechanisms that underlie these pathologies, despite their global impact on the population and the undeniable need for optimized treatment.

We collected in IntAct a list of interactions between microRNAs and genes associated with the onset or progression of rare disorders. The list of genes was retrieved from the Genome England PanelApp (35). Among the genes listed for each disease, few of them have been reported in the literature to bind to microRNAs. We considered several rare diseases (listed in material and methods), and for the present study, we selected three of them, since they have a considerable number of genes regulated by microRNAs: GFEC, mitochondrial disorders (MD) and EOD.

**Figure 4.**
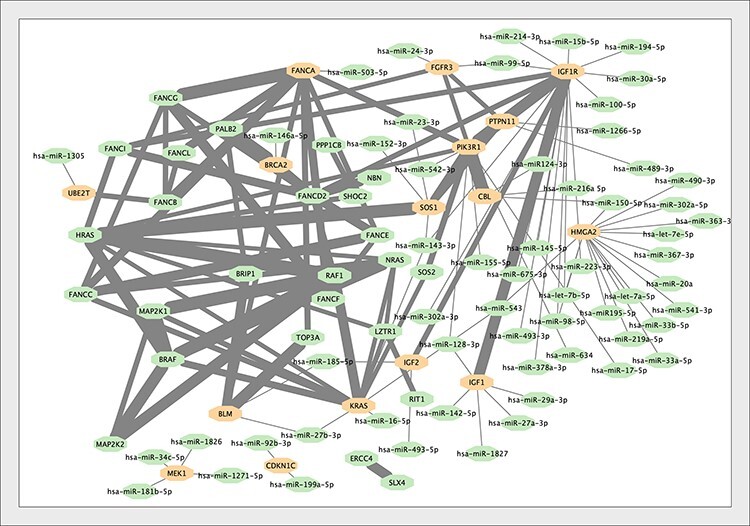
The interaction network of the genes associated with Growth Failure in Early Childhood (GFEC). Interactions within proteins associated with GFEC merged into the microRNA–mRNA network. Proteins regulated by microRNAs are indicated. The thickness of the edge between proteins is proportional to the MI score (i.e. reliability) of the interaction (24).

Table 1 shows the list of the genes that were found to interact with microRNAs and annotated in IntAct. GFEC is influenced by 48 genes, 17 of which were annotated in IntAct for mRNA–microRNA interactions (Figure 4). Approximately 230 genes are associated with mitochondrial disorders (plus a few mitochondrial tRNA genes we did not consider). We found microRNA interactions suitable to be annotated in IntAct only for 14 of these. Finally, a list of 27 genes is associated with EOD, 13 of which were annotated in IntAct.

**Table 1.** Genes associated with the rare diseases and regulated by microRNAs

EOD	GFEC	Mitochondrial disorders
APP	BLM	AIFM1
ATXN1	BRCA2	COX10
CSF1R	CBL	DMPK
DNMT1	CDKN1C	DNM2
GRN	FANCA	ISCU
HTT	FGFR3	MFN2
ITM2B	HMGA2	MPC1
MAPT	IGF1	NDUFA10
NOTCH3	IGF1R	PDHB
PRNP	IGF2	PDHX
PSEN1	KRAS	SLC25A1
TARDBP	MAP2K1 (MEK1)	SLC25A12
TBK1	OBSL1	TAZ
	PIK3R1	UQCC2
	PTPN11	
	SOS1	
	UBE2T	

Only interactions confirmed by mutagenesis analysis are considered.

We used the BINGO tool to evaluate biological processes statistically over-represented in the microRNA-regulated genes. We found an enrichment of genes involved in positive regulation of cellular processes and cell proliferation, as well as proteins involved in signalling pathways, in genes associated with GFEC and EOD, in relation to non-annotated genes (Supplementary Table 2).

A small number of genes associated with mitochondrial dysfunctions are regulated by microRNAs, and the enrichment analysis did not show significant enrichment for any term. Interestingly, however, there is a strong enrichment of specific metabolic and biosynthetic processes, such as generation of precursor metabolites, ATP synthesis coupled to electron transport, oxidative phosphorylation and tRNA metabolic processes, in genes associated with MD and not regulated by microRNAs (Supplementary Table 2).

We compared our results with two other databases: miRTarBase (interactions confirmed by ‘Luciferase assay’) and RAID (interactions confirmed by ‘strong evidence’). The low percentage of microRNA-regulated genes was confirmed in the two datasets: in Supplementary Table 1, the number of interacting microRNAs for each gene in the three databases is reported, and most of the genes appear to have no described interactors. Networks resulting from the integration and the intersection of the three dataset are shown in Supplementary Figure 1.

**Figure 5.**
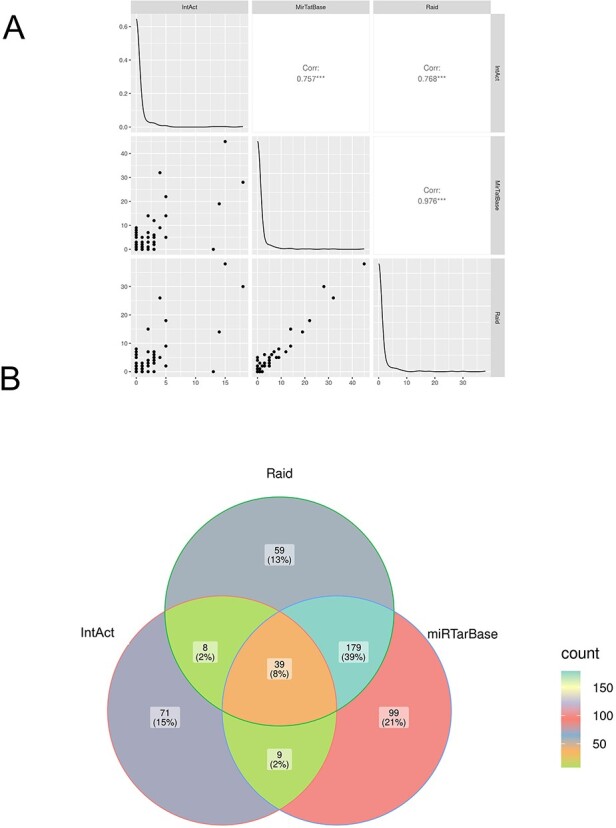
Comparison of microRNA–mRNA interactions annotated in IntAct, MirTarBase and RAID. (A) Correlation analysis: the number of microRNA-regulating genes associated with the diseases in the three datasets was compared (see also Supplementary Table 1). (B) Venn diagram comparing interacting pairs of microRNA–mRNA annotated in the three datasets. The Jaccard similarity coefficient (in parentheses) is calculated as intersection over union and indicates that only 8% of the interactions are annotated in all the datasets and that each resource contains some exclusive information.

Interestingly, there is a very high correlation among the number of interacting microRNAs annotated in the three databases for each gene (Figure 5A). This suggests that, although the coverage of annotated microRNA interactions may still be low, the networks reflect what is currently known in the literature, with more than half genes of each list not affected by microRNA regulation, many involved in one or few interactions and a small number targeted by a huge number of microRNAs (Supplementary Figure 2, Supplementary Table 3). This observation does not imply that those genes are certainly not regulated by microRNAs, but that the present literature does not contain evidence of direct binding. The lower correlation of the IntAct database with the other two is explained with the consideration that we have selected luciferase experiments only when validated with mutagenesis.

Despite the high correlation, the three datasets do not contain the same interactions: the Venn diagram shows that only 8% of them (calculated from the union of the three datasets) are present in all databases, while each dataset contains 21%, 15% or 13% of interactions not annotated in the others (Figure 5B). This suggests that a combined effort of different resources is necessary to get the full picture and to help advance research in gene regulation (Supplementary Figure 1).

### Discussion

Investigations of gene-disease associations typically focus on protein variants; however, as the relevance of ncRNAs becomes more evident, their involvement in many common disorders is conceivable. For an accurate comprehension of microRNA functions, we need to assemble the entire set of miRNA–mRNA interactions, possibly annotated using standard procedures that are clearly explained to the user. Some resources collect microRNA target interactions using natural language processing to find co-occurrences of terms, followed by manual review. Despite the undeniable relevance and high coverage of these resources, there is no general acceptance of what to consider a bona fide interactor, versus an indirect regulator. The reliability of microRNA interaction data has been extensively discussed in (15, 36–38). We have filtered the experimental data according to high-quality standards to collect a set of reliable microRNA targets. The IntAct database contains approximately 900 interactions involving more than 200 microRNAs. Currently being modest coverage, the dataset is expected to increase, as new interactions are continually being added.

The target mRNAs were annotated with the Ensembl transcript ID (26) as the identifier. In order to expedite the integration with other data identified by gene identifiers, the entries are also linked to the Ensembl gene ID. Referring to a specific transcript sequence allows the user to unequivocally annotate the interacting regions, confirmed by the mutagenesis analysis.

Recent advances in RNA molecule stabilization and delivery methods have renewed interest in RNA-based therapies. Chemical modifications of the nucleotides increase their resistance to nucleases, and encapsulation of the microRNA mimics in neutral lipids allows the delivery of the molecules into living cells or organisms (39). In the last years, several microRNA-based therapeutics have been developed and some have entered phase II or III of clinical trials (40). The availability of exhaustive information about microRNA binding could accelerate the design of specific mimics or inhibitors and predict the effect of the perturbation on the neighbourhood interactors. Since RNA–RNA interactions occur through base pairing, the design and development of interfering molecules is easier than protein inhibitors, as demonstrated by the common use of complementary molecules in scientific papers.

We have prioritized the annotation of microRNA-regulating genes involved in rare diseases, hopefully contributing to filling the gap of knowledge in this field. It is worth mentioning that the IntAct database has already collected a dataset of protein–protein interactions directly involved in rare diseases (27). Some diseases are directly associated with genetic mutations or variants: the disease is a direct consequence of the mutation, or, more frequently, the mutation increases the probability of the onset or progression. When the mutation directly affects a PPI interaction, the information can be retrieved from the database (41). Very few papers describe the role of microRNA in rare diseases, and hopefully, the list of interactions that affect genes associated with the diseases may help in elucidating their role. Although ‘rare’, the ‘rare diseases’ affect more than 30 million people in Europe and are considered one of the major public health issues. As the return of investment in research on each individual disease may be limited, no treatment and diagnostic tests have been developed for many of them. The annotation of genes involved in rare diseases has been recognized as a crucial step to expedite the diagnosis and to establish the appropriate treatment (34). We have expanded this knowledge by collecting existing data on the regulation of genes by microRNAs.

Among the analysed genes, seven involved in GFEC and three involved in EOD seem to be hubs in the microRNA–mRNA network, regulated by more than 10 microRNAs (Supplementary Figure 2). In particular, PRNP, DNMT1 and APP play a role not only in the EOD but also in several other forms of dementia and brain diseases.

To our knowledge, no other collections of binding regions experimentally validated with mutation analysis and mapped on stable entries are available for microRNA targets. Mutations with no effect are also recorded, when available. This information can be compared with disease-related genomic variants to highlight relevant microRNA gene regulation events. The IntAct resource offers an ideal environment to store and re-use the information, as it will update entry identificatives and cross-references to keep them up-to-date with Uniprot and other resources. Data are available not only from the database web interface but also from Psicquic view (http://www.ebi.ac.uk/Tools/webservices/psicquic/view/home.xhtml), IntAct App (28) and externally accessible API (27).

In the field of RNA therapeutics, the accessibility to collections of ‘true’ interactors and the availability of high-reliable networks will simplify procedures to assess the value and potential unrelated effects of a therapeutic product and will help the design of competitors.

## Supplementary Material

baad066_Supp

## Data Availability

The data underlying this article are available in the IntAct database (https://www.ebi.ac.uk/intact/).
