# Correction to: The landscape of microRNA interaction annotation: analysis of three rare disorders as a case study

**DOI:** 10.1093/database/baae131

**Published:** 2025-01-13

This is a correction to: Simona Panni, Kalpana Panneerselvam, Pablo Porras, Margaret Duesbury, Livia Perfetto, Luana Licata, Henning Hermjakob, Sandra Orchard, The landscape of microRNA interaction annotation: analysis of three rare disorders as a case study, *Database*, Volume 2023, 2023, baad066, https://doi.org/10.1093/database/baad066

In the originally published version of this manuscript, the first author’s name was presented incorrectly—the correct name is Simona Panni.

This error has been corrected.

