# Supplementary material for: The landscape of microRNA interaction annotation: analysis of three rare disorders as a case study
Source: Database (Oxford). 2023 Oct 11;2023:baad066. doi: 10.1093/database/baad066 (PMC10566539; doi:10.1093/database/baad066)
Supplement: baad066_Supp [file baad066_supp.zip › suppl_data/Supplementary Table 3.docx]

| Network Parameters | GEFC network | MD network | EOD network |
| --- | --- | --- | --- |
| Number of nodes | 222 | 93 | 93 |
| Number of edges | 306 | 69 | 92 |
| Avg number of neighbors | 2,8 | 2 | 2,1 |
| Avg shortest Path lenght | 3,7 | 2,5 | 4 |
| Network diameter | 9 | 5 | 7 |
| Network radius | 5 | 3 | 4 |
| Network density | 0,013 | 0,016 | 0,021 |
| Network centralization | 0,314 | 0,39 | 0,26 |
|  |  |  |  |

**Supplementary Table 3** General Network properties for Growth Failure in Early Childhood (GEFC), Mitochondrial Disorders (MD) and Early Onset Dementia (EOD) merged networks**. I**ntegrated networks were analysed with Cytoscape analyzing tool [29]
