# Supplementary figures and images for: The landscape of microRNA interaction annotation: analysis of three rare disorders as a case study
Source: Database (Oxford). 2023 Oct 11;2023:baad066. doi: 10.1093/database/baad066 (PMC10566539; doi:10.1093/database/baad066)

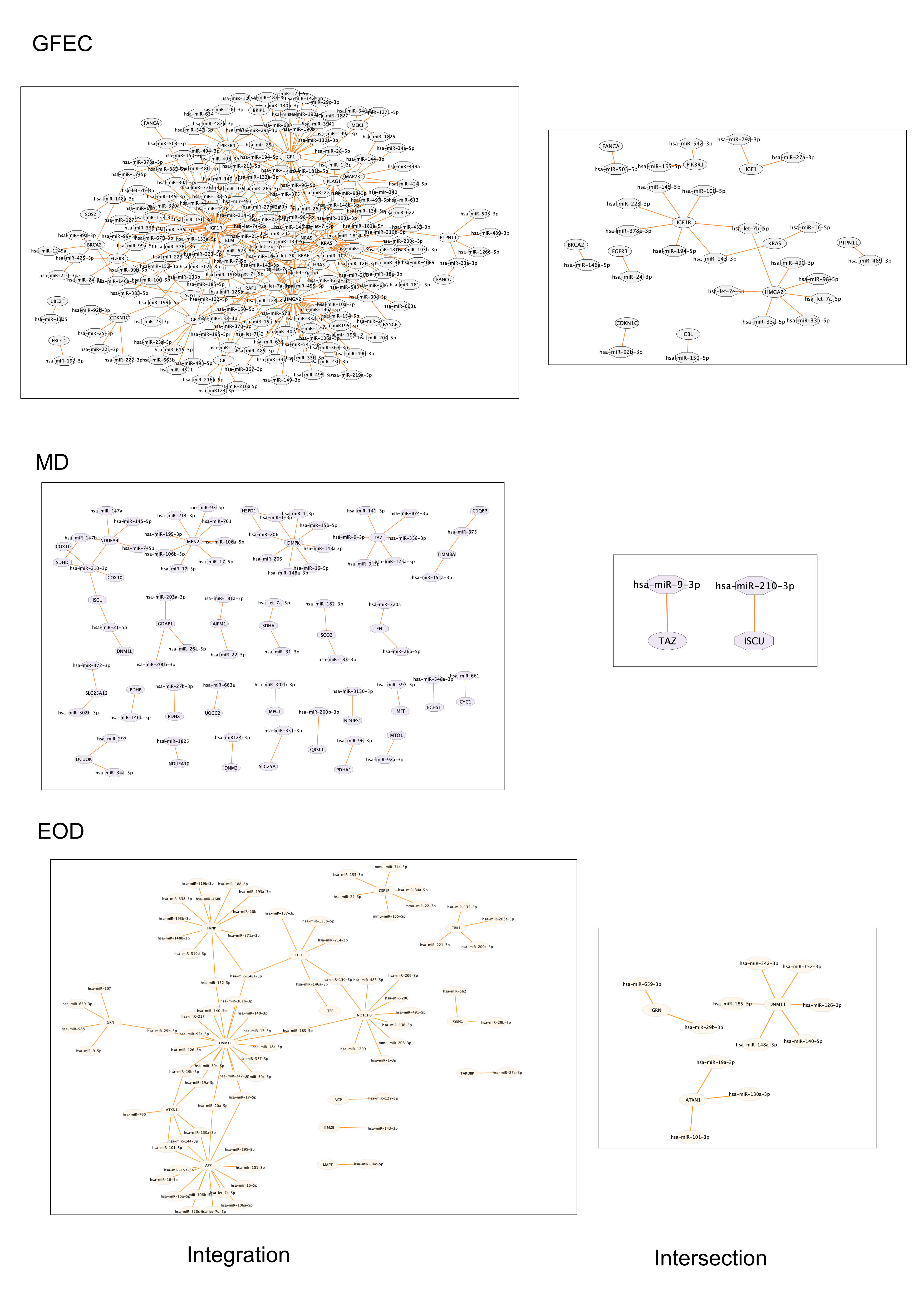

Supplement: baad066_Supp [file baad066_supp.zip › suppl_data/Supplementary Figure 1.jpg]

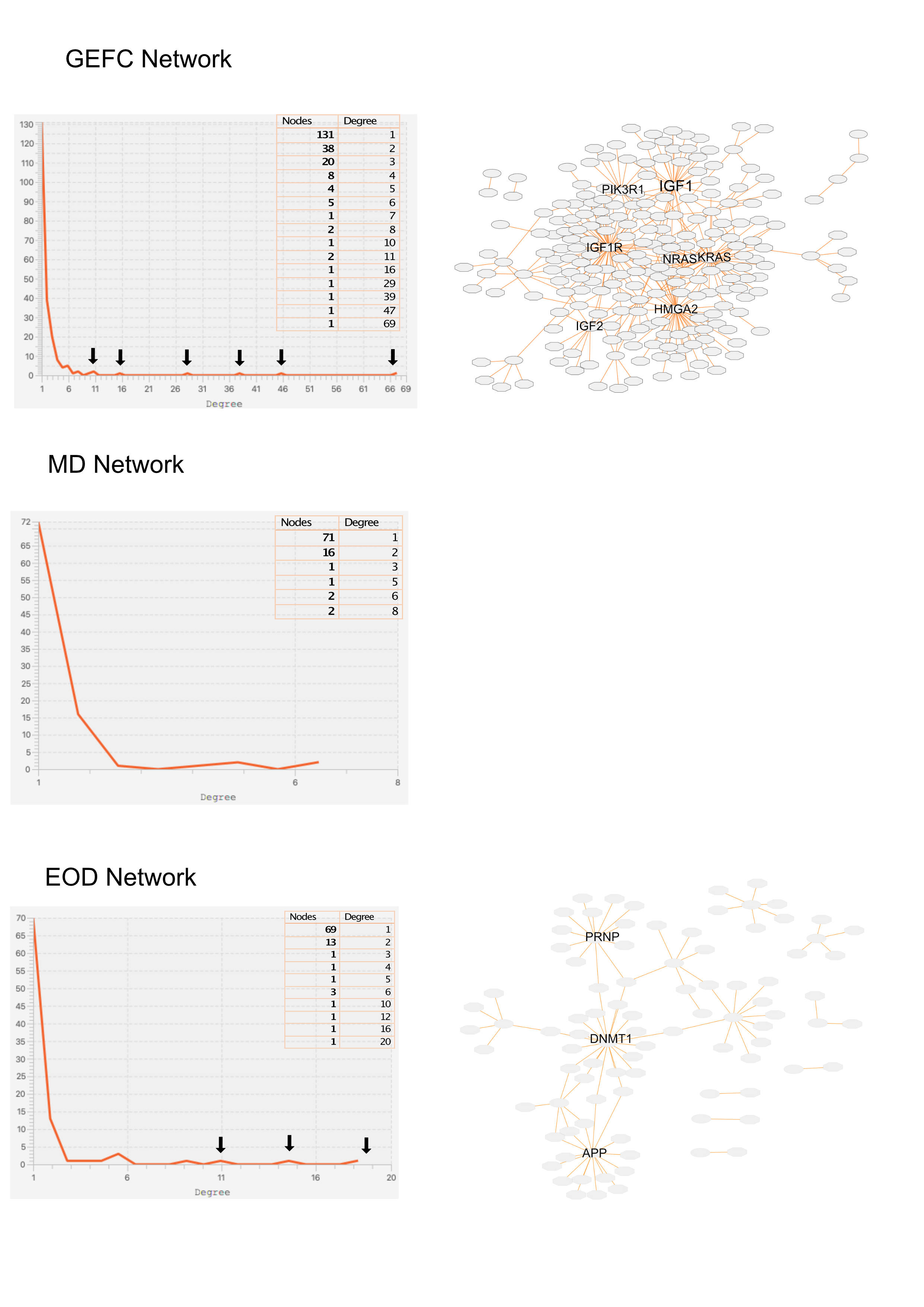

Supplement: baad066_Supp [file baad066_supp.zip › suppl_data/Supplementary Figure 2.jpg]
